# Combined Immersive and Nonimmersive Virtual Reality With Mirror Therapy for Patients With Stroke: Systematic Review and Meta-Analysis of Randomized Controlled Trials

**DOI:** 10.2196/73142

**Published:** 2025-10-10

**Authors:** Chaoran Gao, Yuan Chen, Yixin Wei, Yitong Qiu, Huiyan Song, Chenfan Gui, Qiang Gao

**Affiliations:** 1 Department of Rehabilitation Medicine, West China Hospital, Sichuan University, 37 Guoxue Alley, Chengdu, 610041, China, 86 18980605992; 2 Key Laboratory of Rehabilitation Medicine in Sichuan Province, West China Hospital, Sichuan University, Chengdu, China; 3 Department of Pharmacy, Evidence-Based Pharmacy Center, West China Second Hospital, Sichuan University, Chengdu, China

**Keywords:** mirror therapy, virtual reality, stroke, upper extremity, hand, systematic review, meta-analysis, rehabilitation

## Abstract

**Background:**

Stroke frequently leads to various functional impairments. Both virtual reality (VR) and mirror therapy (MT) have shown efficacy in stroke rehabilitation. In recent years, the combination of these 2 approaches has emerged as a potential treatment for patients with stroke.

**Objective:**

This study aimed to evaluate the efficacy of combined immersive and nonimmersive VR with MT in stroke rehabilitation.

**Methods:**

Five electronic databases were systematically searched for relevant papers published up to January 2025. Randomized controlled trials (RCTs) that investigated the combination treatment of VR and MT for patients with stroke were included. A gray literature search was also conducted. The risk of bias and the certainty of the evidence were assessed using the Cochrane Collaboration’s tool and the Grading of Recommendations, Assessment, Development, and Evaluation (GRADE) guidelines, respectively.

**Results:**

A total of 475 patients from 14 RCTs were included, of which 7 were eligible for meta-analysis. Meta-analysis revealed significant improvements in upper extremity (UE) motor function and hand dexterity, as evidenced by the Fugl-Meyer Assessment–Upper Extremity (FMA-UE; mean difference, MD 3.50, 95% CI 1.47 to 5.53; *P*=.<001), the manual function test (MD 2.15, 95% CI 1.22 to 3.09; *P*<.001), and the Box and Block Test (MD 1.09, 95% CI 0.14 to 2.05; *P*=.03). Subgroup analyses based on disease duration (>6 months or not) revealed significant differences in the FMA-UE outcome. However, the pooled FMA-UE improvement did not consistently exceed the established minimal clinically important difference (4.25‐7.25), indicating that while statistically significant, the clinical significance of the observed effect remains uncertain. Narrative evidence also suggested potential benefits for lower extremity function, dynamic balance, and quality of life, though these findings were not meta-analyzed and should be interpreted with caution.

**Conclusions:**

Moderate-quality evidence supports combined VR and MT as a promising nonpharmacological intervention to improve upper extremity function and hand dexterity in stroke rehabilitation. While the intervention demonstrates statistically significant effects, it does not reach the minimum clinically important difference for the FMA-UE outcome. Preliminary descriptive evidence indicates possible advantages for lower extremity function, balance, and quality of life.

## Introduction

### Background

Stroke is one of the leading causes of disability, significantly impairing patients’ quality of life (QoL) [1]. It can lead to a range of functional impairments, including motor dysfunction [2], cognitive dysfunction [3], and speech dysfunction [4]. Given the global prevalence and profound impact of stroke [5], there is an urgent need to improve health care services and rehabilitation strategies.

Mirror therapy (MT) is a rehabilitation method based on the theory of “mirror neurons,” which are activated both when individuals execute a given motor action and when they merely observe others performing the identical action [6]. First demonstrated by Ramachandran in the 1990s, MT was initially used for the treatment of phantom limb pain [7] and hemiparesis [8]. Subsequently, MT has been widely adopted in stroke rehabilitation. Several systematic reviews and meta-analyses have demonstrated the efficacy of MT in enhancing upper extremity (UE) and lower extremity (LE) motor function, balance, gait, pain, and activities of daily living (ADL) [9-11]. In addition, MT has also been shown to be effective in promoting cognitive and speech function recovery [12,13]. Consequently, MT can be considered a complementary treatment to conventional therapy for patients with stroke.

With advancements in technology, virtual reality (VR) has emerged as a prevalent therapeutic approach in stroke rehabilitation, owing to its potential to enhance patient motivation, adaptability, and variability based on patient baseline, as well as to reduce medical costs [14]. Similar to MT, VR therapy has been shown to improve UE gross motor function and independence in daily life [15]. It has also demonstrated benefits for LE motor function, balance, gait, and cognition [16] in patients with stroke. The mechanisms of VR effects in stroke rehabilitation are related to VR-induced changes in neural plasticity and the positive correlations between neural plasticity changes and functional recovery [17]. In stroke rehabilitation, VR systems are generally classified into 2 types: immersive virtual reality (IVR) and nonimmersive virtual reality (NIVR). NIVR is typically experienced in a 2D environment and presented via devices such as computer screens or gaming consoles [18]. Users interact with the displayed environment using input devices such as a mouse, joysticks, CyberGloves, CyberGrasps, or force sensors [19]. An IVR system typically includes a head-mounted display and 3D interaction devices (eg, VR controllers or motion-tracking gloves) that enable users to engage with the virtual environment. Unlike NIVR, IVR offers a fully immersive experience, allowing users to interact dynamically with the virtual environment—a defining feature of immersive VR technologies [20].

Recent research suggests that combining VR and MT may offer additional benefits in stroke rehabilitation. For instance, Okamura et al have demonstrated that this combined therapy can significantly improve UE function following a stroke [21]. Although several reviews have confirmed the effectiveness of MT or VR in stroke rehabilitation, fewer have investigated the synergistic effects of combining these 2 approaches. Moreover, many of these reviews have experienced methodological shortcomings, such as the direct analysis of postintervention data rather than changes from pre- to post-intervention, which may lead to biased conclusions. In response to these limitations, we undertook this systematic review and meta-analysis. In this study, we defined the combination treatment of VR and MT as VR-based MT, referring to the integration of core MT principles (eg, symmetrical movement observation via a mirror or mirrored virtual images) into VR platforms. Furthermore, we also included studies that applied VR and MT sequentially, such as those administering VR interventions followed by MT sessions.

### Objective

Based on this background, the overall objective of this study was to evaluate the efficacy of combining immersive and nonimmersive VR with MT as a rehabilitation strategy for patients with stroke. We hypothesized that a systematic review and meta-analysis of RCTs would yield robust and comprehensive evidence supporting the efficacy of combined VR and MT in stroke rehabilitation.

## Methods

### Overview

This systematic review adhered to the PRISMA (Preferred Reporting Items for Systematic Reviews and Meta-Analyses) guidelines [22]. The review protocol was prospectively registered in the PROSPERO database (CRD42024572150).

### Search Strategy

The literature search was conducted across the scientific databases PubMed, Embase, Web of Science, Cochrane, and Scopus up to January 2025. No language or publication date filters were applied during the search. Keywords and Medical Subject Headings descriptors, including “stroke,” “virtual reality,” and “mirror therapy,” combined using Boolean operators “AND” and “OR,” were used to structure the search strategy, which is detailed in Multimedia Appendix 1. An additional search of the gray literature was conducted using Google Scholar, ProQuest Dissertations and Theses, the Bielefeld Academic Search Engine, and the China National Knowledge Infrastructure on the same day. No restrictions on language or publication date were applied.

### Inclusion and Exclusion Criteria

The PICOS (population, intervention, comparison, outcome, and study design) framework [23] was used to delineate the inclusion criteria: (1) patients diagnosed with stroke (population); (2) the combination of VR and MT, defined as integrating core principles of MT into the VR platform or applying VR and MT sequentially. The VR intervention includes 2 main forms: IVR and NIVR. No restrictions were applied regarding specific VR platforms or MT setups (intervention); (3) any other therapy or control group, including MT alone, sham therapy, occupational therapy (OT), and conventional therapy (comparison); (4) stroke-related outcomes assessed at pre- and post-intervention timepoints, such as UE and LE motor function, hand dexterity, and balance (outcome); and (5) randomized controlled trials (RCTs) (study design).

Exclusion criteria for this review included: (1) the presence of comorbidities in patients; (2) studies that did not report mean and SD values of outcome changes required for effect size calculations; (3) data could not be imputed based on the information available in the publication; (4) data not obtainable within 1 month of contacting the corresponding authors; or (5) review studies, case reports, or abstracts.

### Study Selection

Two reviewers independently executed the search, adhering to the PRISMA guidelines throughout the entire screening and selection procedure. Discrepancies were resolved through consultation with a third reviewer. The screening and selection process comprised the following stages: (1) removal of duplicate records, (2) screening titles and abstracts for relevance, and (3) full-text review of the remaining studies to determine their eligibility based on the predetermined inclusion or exclusion criteria.

### Data Extraction

Data extraction was conducted in accordance with the PRISMA guidelines by 2 independent reviewers. The following data were extracted from each paper: author, publication year, sample size, patient characteristics (sex, age, and disease duration), intervention details (type, duration, and frequency), and stroke-related outcomes related to stroke (eg, UE and LE motor function, hand dexterity, and balance). For some trials (eg, Choi et al [24], Hsu et al [25], Jo et al [26] that included multiple intervention arms, we selected the MT group as the control group, since all these studies used this control measure. Should conflicts arise, a third reviewer was involved to resolve them. In cases where data were insufficient, the corresponding author was contacted via email to request additional information.

### Assessment of Risk of Bias and Certainty of Evidence

The risk of bias in the included trials was assessed using the Cochrane Collaboration tool focusing on the following domains: random sequence generation, allocation concealment, blinding of participants and health care providers, blinding of outcome assessors, incomplete outcome data, selective reporting, and other sources of bias, including significant differences between study groups at baseline and different intervention durations between study groups [27]. The Review Manager software (RevMan version 5.4; The Cochrane Collaboration) was used for this review. The software provides a structured description and assessment for each item, with predefined responses that categorize the risk of bias as low (indicated by “yes”), high (indicated by “no”), or unclear (indicating a lack of information or uncertainty regarding potential bias). The assessment was conducted by 2 independent authors, and any discrepancies were addressed by a third reviewer to mediate conflicts as they arose.

The Grading of Recommendations, Assessment, Development, and Evaluation (GRADE) approach was used by 2 independent reviewers to assess the certainty of the evidence. The GRADE approach is a widely recognized tool for grading evidence certainty in systematic reviews and clinical practice guidelines. It categorizes evidence certainty into 4 levels: “very low,” “low,” “moderate,” or “high,” each determined by specific criteria [28]. Factors that may diminish evidence certainty (eg, risk of bias, inconsistency, indirectness, imprecision, and publication bias) as well as those that may enhance it (eg, large effect, plausible confounding, and dose-response) were evaluated [29]. In the event of discrepancies in the certainty ratings among the reviewers, a third reviewer was consulted.

### Data Analysis

Outcomes were included in the meta-analysis if they were reported in more than 2 trials. Statistical analysis and the generation of forest plots for result visualization were performed using RevMan. Relevant statistical measures, including means and SDs of differences between pre- and post-intervention and sample sizes, were extracted and then imported into RevMan. For studies that reported only pre- and post-intervention means and SDs, the mean and SD of the change scores were calculated as follows:

Mean_change_=Mean_post_ − Mean_pre_

$SD_{change}=\sqrt{SD_{pre}^{2}+\;SD_{post}^{2}-\;2\;\times \;r\;\times \;SD_{pre}\times \;SD_{post}}$

where r is the correlation coefficient between pre- and post-intervention values. If the correlation was not available in the original publication, a value of *r*=0.5 was assumed according to the Cochrane Handbook for Systematic Reviews of Interventions [30].

For studies reporting medians and IQRs, the data were transformed into means and SDs as follows: (1) mean = median and (2) SD = IQR/1.35 [31]. Mean differences (MDs) were used as effect sizes because the included studies applied the same or similar outcome measures with consistent units, with 95% CIs reported. Standardized mean differences were not used. The inverse variance method was applied for continuous variables. Heterogeneity among the trials was evaluated through the chi-square test and *I*^2^ statistics, with thresholds of 25%, 50%, and 75% defining low, moderate, and high levels of heterogeneity, respectively [32]. A fixed-effects model was adopted to estimate the pooled effect size. In cases of substantial heterogeneity (*I*²>50%), a random-effects model was used. Subgroup analyses were performed to investigate potential associations between outcomes and disease durations. Outliers in the meta-analysis were identified using studentized leave-one-out sensitivity analyses [33]. The Egger regression test was used to measure the possibility of publication bias, with 2-tailed *P* values of <.05 indicating potential publication bias [34]. Sensitivity analyses and the Egger regression test were performed using Stata (version 17; StataCorp LLC).

### Ethical Considerations

This study is a systematic review and meta-analysis based on previously published studies. No new patient data were collected, and no human participants were directly involved. Therefore, institutional review board approval and informed consent were not required. The review process adhered to the PRISMA (Preferred Reporting Items for Systematic Reviews and Meta-Analyses) guidelines, and the study protocol was prospectively registered in PROSPERO. All included studies were cited appropriately, and no conflicts of interest were reported that could have influenced the conduct of this review.

## Results

### Study Selection Process

**Figure 1.**
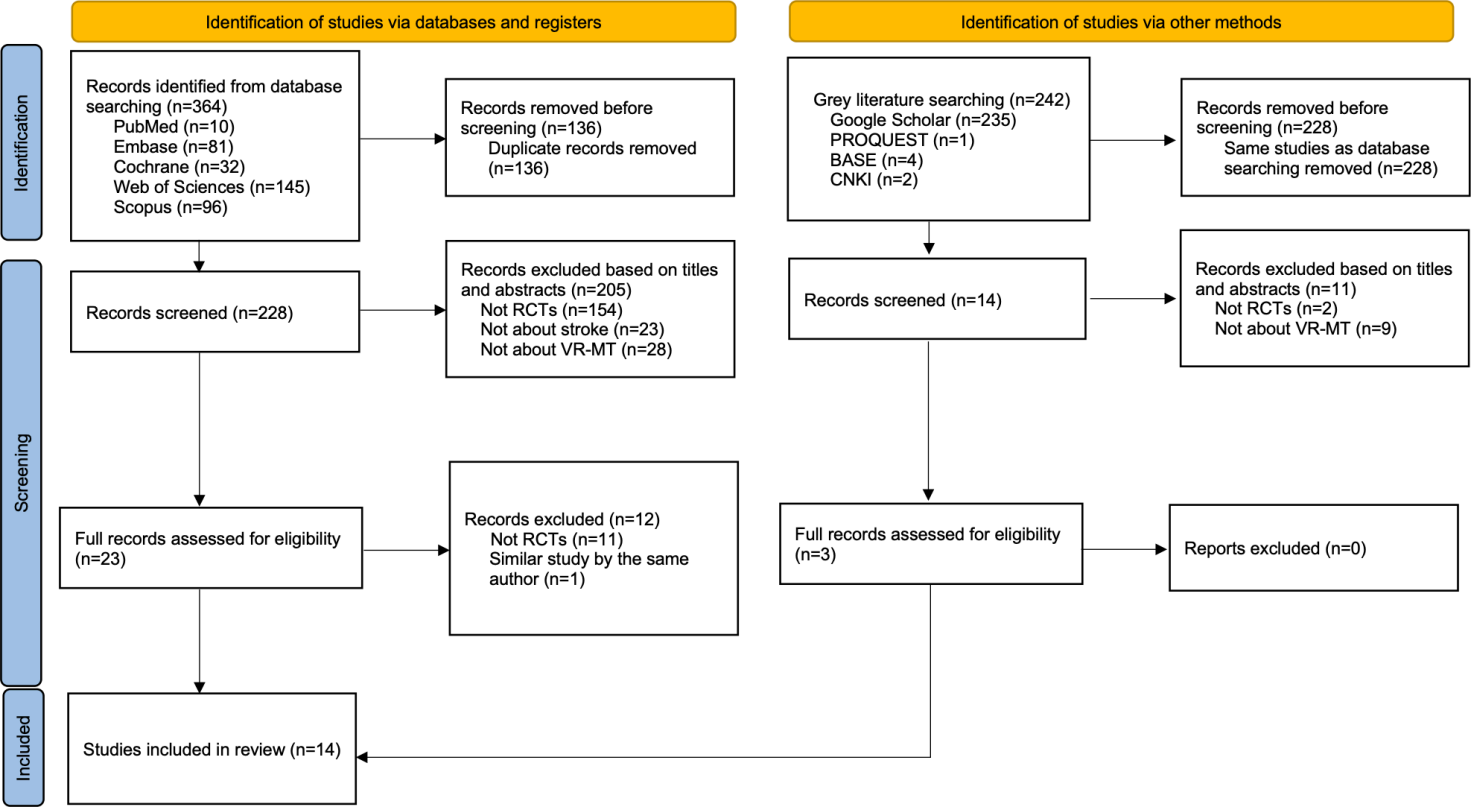
Study selection process. BASE: Bielefeld Academic Search Engine; CNKI: China National Knowledge Infrastructure; MT: mirror therapy; RCT: randomized controlled trial; VR: virtual reality.

The initial literature search yielded 364 papers, of which 136 (37.4%) were duplicates and excluded. From the remaining 228 papers after excluding duplicates, 205 (89.9%) were removed because they were irrelevant to the study objectives. After further exclusion of 12 nonRCTs and 1 duplicate study, a total of 11 RCTs were included in the current systematic review [24-26,35-42]. In addition, 3 RCTs were identified through gray literature searching [43-45]. Figure 1 shows the study selection process and reasons for exclusion.

### Characteristics of the Included Trials

The 14 RCTs [24-26,35-45] included in the review were all published in English, spanning from 2012 [35] to 2024 [26]. These trials were conducted across Asia (China [25,37,38,43,44] and the Republic of Korea [24,26,35,42], North America (the United States [36]), and Europe (Romania [39], the United Kingdom [40], Poland [41], and Liechtenstein [45]), involving a total of 475 participants (experimental group: n=244, 51% and control group: n=231, 49%). Among the included studies, Morgenstern [45] reported the highest number of participants (n=80), while Strong et al [40] had the smallest sample size (n=7). The detailed characteristics of the included trials are shown in Table 1.

**Table 1.** Characteristics of the RCTs included in the systematic review and meta-analysis.

Study	Number of participants, n (male and female)	Sample size and age (years), mean (SD)	Disease duration	Intervention of EG^a^	Intervention of CG^b^	Duration and frequency of intervention	Outcome	Measuring instrument
In et al, 2012 [35]	19 (11 and 8)	EG: 11 (63.5 [11.8]); CG: 8 (64.5 [12.7])	>6 months	VR^c^ + MT^d^	ST^e^	30 min/d, 5 d/wk, 4 weeks	UE motor function, muscle tension, and hand dexterity	FMA-UE^f^ (total), MAS^g^-UE, BBT^h^, JHFT^i^, and MFT^j^
In et al, 2016 [42]	25 (15 and 10)	EG: 13 (57.3 [10.5]); CG: 12 (54.4 [11.4])	>6 months	VR + MT	ST	30 min/d, 5 d/wk, 4 weeks	Balance and gait	BBS^k^, FRT^l^, TUGT^m^, postural sway, and 10 mWV^n^
Choi et al, 2019 [24]	24 (14 and 10)	EG: 12 (58.0 [15.2]); CG: 12 (59.6 [11.9])	>6 months	VR + MT	MT	30 min/d, 3 d/wk, 5 weeks	UE motor function, neck discomfort, and QoL^o^	MFT, NDS^p^, and SF-8^q^
Bullock et al, 2020 [36]	14 (4 and 10)	EG: 7 (N/A); CG: 7 (N/A)	N/A	VR + MT	ST	5‐20 min/session, 8 sessions	Degree of disability	OHS^r^
Lin et al, 2021 [37]	18 (13 and 5)	EG: 9 (49.7 [13.4]); CG: 9 (58.8 [9.6])	>6 months	VR + MT	MT	50 min/d, 2 d/wk, 9 weeks	UE motor function	FMA-UE (total, shoulder, elbow and forearm, wrist, hand, and coordination)
Mekbib et al, 2021 [38]	23 (17 and 6)	EG: 12 (52.2 [13.3]); CG: 11 (61.0 [7.7])	<3 months	VR + MT	OT	120 min/d, 4 d/wk, 2 weeks	UE motor function, ADL^s^, and imaging results	FMA-UE (total), BI^t^, and rs-fMRI^u^
Miclaus et al, 2021 [39]	59 (15 and 44)	EG: 31 (59.0 [10.1]); CG: 28 (60.7 [8.2])	>6 months	VR + MT	CT	70 min/d, 10 days	Muscle strength, ROM^v^, stroke severity, ADL, muscle tension, LE motor function, and balance	ROM-LE, MMT^w^, FMA-LE^x^, MRS^y^, FIM^z^, MAS-LE, and FRT
Strong et al, 2021 [40]	7 (3 and 4)	EG: 4 (46.0 [N/A])^aa^; CG: 3 (53.7 [NA])	>3 months	VR + MT	9HPT^ab^ training	45 min/d, 4 d/wk, 4 weeks	Finger dexterity and feasibility	9HPT, IMI^ac^, and SUS^ad^
Hsu et al, 2022 [25]	35 (15 and 20)	EG: 18 (52.9 [11.8]); CG: 17 (56.7 [11.5])	>6 months	VR + MT	MT	50 min/d, 2 d/wk, 9 weeks	UE motor function, hand dexterity, hand touch-pressure threshold, and muscle tension	FMA-UE (total, shoulder, elbow and forearm, wrist, hand, and coordination), MAL^ae^, BBT, SWM^af^, and MAS (wrist and hand)
Morgenstern, 2022 [45]	80 (41 and 39)	EG: 40 (65.5 [6.7]); CG: 40 (64.8 [7.5])	N/A	VR + MT	CT	N/A	Spatial neglect severity and UE motor function	BIT^ag^ and ARAT^ah^
Wang, 2022 [44]	60 (46 and 14)	EG: 31 (53.5 [13.1]); CG: 29 (56.5 [18.3])	<6 months	VR + MT	CT	60 min/d, 5 d/wk, 2 weeks	UE motor function and cognitive function	FMA-UE (total), ROM-UE, Brunnstrom-UE, MMSE^ai^, MOCA^aj^, and TMT^ak^
Xu, 2022 [43]	61 (34 and 27)	EG: 31 (56.6 [7.4]); CG: 30 (58.8 [6.3])	>1 month to <12 months	VR + MT	MT	30 min/d, 5 d/wk, 3 weeks	LE motor function and balance	FMA-LE, BBS, TUGT, locus length of gravity, and difference in weight distribution
Sip et al, 2023 [41]	20 (N/A)	EG: 10 (54.9 [4.0]); CG: 10 (59.2 [4.3])	<12 months	VR + MT	MT	30 min/d, 18 days	UE motor function, QoL, and pain	FMA-UE (total), SF-36^al^, and UE pain
Jo et al, 2024 [26]	30 (15 and 15)	EG: 15 (51.7 [13.7]); CG: 15 (51.0 [13.0])	<6 months	VR + MT	MT	30 min/session, 3 sessions/wk, 4 weeks	UE motor function and hand dexterity	FMA-UE (total), MFT, and BBT

a EG: experimental group.

b CG: control group.

c VR: virtual reality.

d MT: mirror therapy.

e ST: sham therapy.

f FMA-UE: Fugl-Meyer Assessment–Upper Extremity.

g MAS: Modified Ashworth Scale.

h BBT: Box and Block Test.

i JHFT: Jebsen Hand Function Test.

j MFT: manual function test.

k BBS: Berg Balance Scale.

l FRT: Functional Reach Test.

m TUGT: Timed Up and Go Test.

n 10 mWV: 10-meter walking velocity.

o QoL: quality of life.

p NDS: Neck Discomfort Score.

q SF-8: Short-Form 8.

r OHS: Oxford Handicap Scale.

s ADL: activities of daily living.

t BI: Barthel Index.

u rs-fMRI: resting-state functional magnetic resonance imaging.

v ROM: range of motion.

w MMT: manual muscle testing.

x FMA-LE: Fugl-Meyer Assessment–Lower Extremity.

y MRS: Modified Rankin Scale.

z FIM: Functional Independence Measure.

aa N/A: not available.

ab 9HPT: Nine-Hole Peg Test.

ac IMI: Intrinsic Motivation Inventory.

ad SUS: System Usability Scale.

ae MAL: Motor Activity Log.

af SWM: Semmes-Weinstein Monofilament Test.

ag BIT: Behavioral Inattention Test.

ah ARAT: Action Research Arm Test.

ai MMSE: Mini-Mental State Examination.

aj MOCA: Montreal Cognitive Assessment.

ak TMT: Trail Making Test.

al SF-36: 36-Item Short Form Survey.

### Risk of Bias and Certainty of Evidence

**Figure 2.**
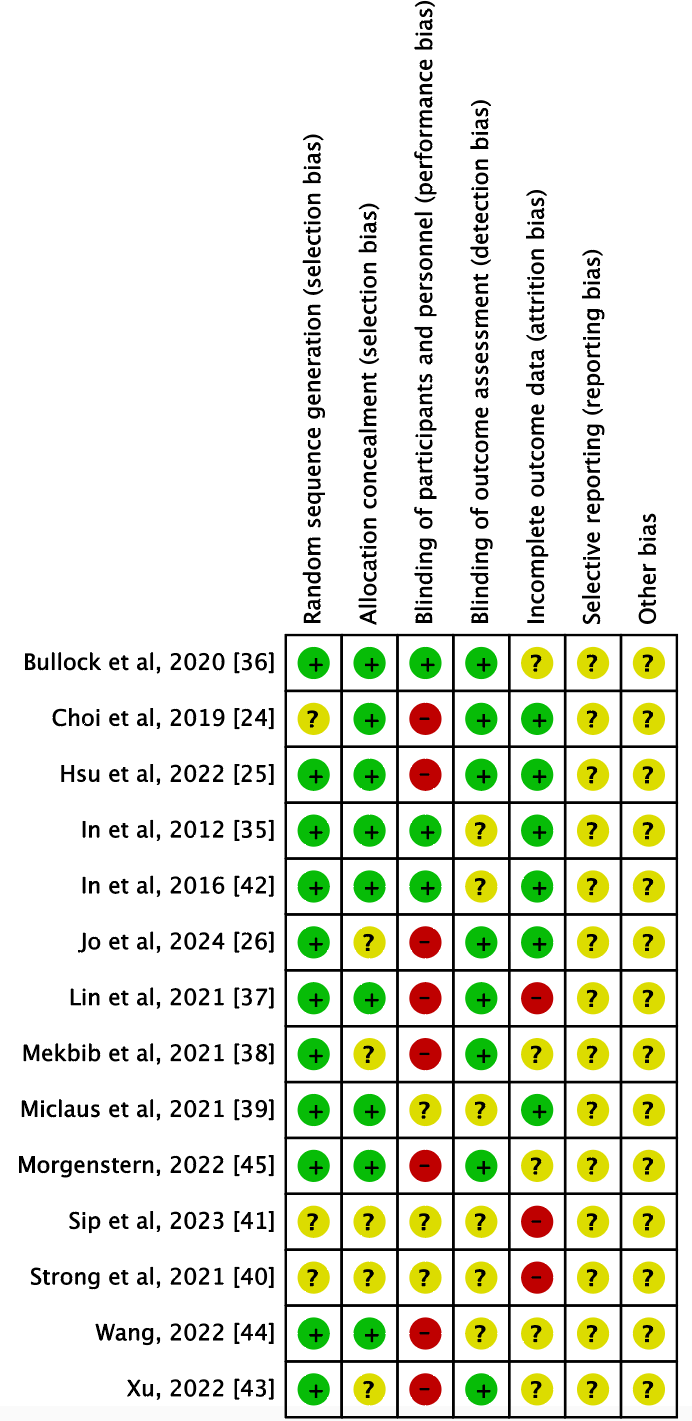
Risk of bias of the studies included in the systematic review [24-26,35-45].

Figures2  3 collectively summarize the risk of bias assessment for the included RCTs, providing both global and individual evaluations for each study. Individual assessments (as shown in Figure 2) revealed that the study by Bullock et al [36] exhibited the lowest risk of bias, while the study by Lin et al [37] presented the highest risk of bias. The greatest risk was noted in the “blinding of participants and personnel” category, which falls under performance bias.

**Figure 3.**
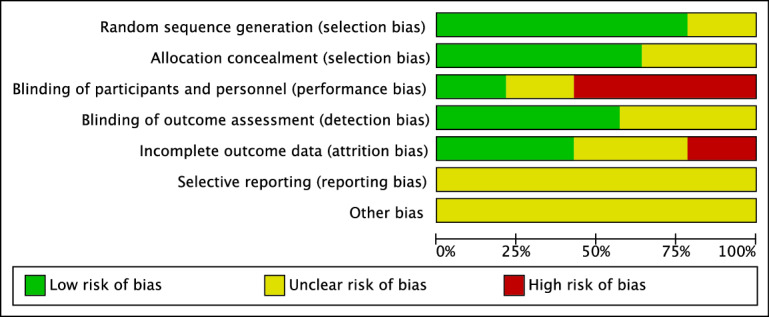
Overall risk of bias, presented as percentage with each category.

Using the GRADE guideline, moderate-quality evidence was found for improvements in UE motor function (Fugl-Meyer Assessment–Upper Extremity [FMA-UE] and manual function test [MFT]) and hand dexterity (Box and Block Test [BBT]). These findings suggest that the true effect is probably similar to the estimated effect. Detailed GRADE assessments are shown in Multimedia Appendix 2. Agreement between the authors was 100% at each stage.

### Synthesis of Results and Meta-Analysis

Of the 14 RCTs, 7 (50%) [24-26,35,37,38,44] with 209 participants (VR+MT: 108 and other treatment: 101) were included in the meta-analysis. The study by Sip et al [41] was not included because the reported FMA-UE (total) results in the experimental group exceeded 66 in both preintervention and postintervention assessments. A low degree of heterogeneity (*I*^2^<50%) was observed across outcomes in the meta-analysis. The main findings of the meta-analysis are shown in Table 2.

**Table 2.** Main meta-analysis findings.

Outcome	Number of included studies	Heterogeneity test	Statistical model	MD^a^ (95% CI)	*z* score	*P* value	Egger test, *P* value
FMA-UE^b^ (total)	6	Low	Fixed effects	3.50 (1.47 to 5.53)	3.39	.0007	.94
MFT^c^	3	Low	Fixed effects	2.15 (1.22 to 3.09)	4.51	<.00001	.85
BBT^d^	3	Low	Fixed effects	1.09 (0.14 to 2.05)	2.24	.03	.60

a MD: mean difference.

b FMA-UE: Fugl-Meyer Assessment–Upper Extremity.

c MFT: manual function test.

d BBT: Box and Block test.

### Effects on UE Motor Function (FMA-UE and MFT)

**Figure 4.**
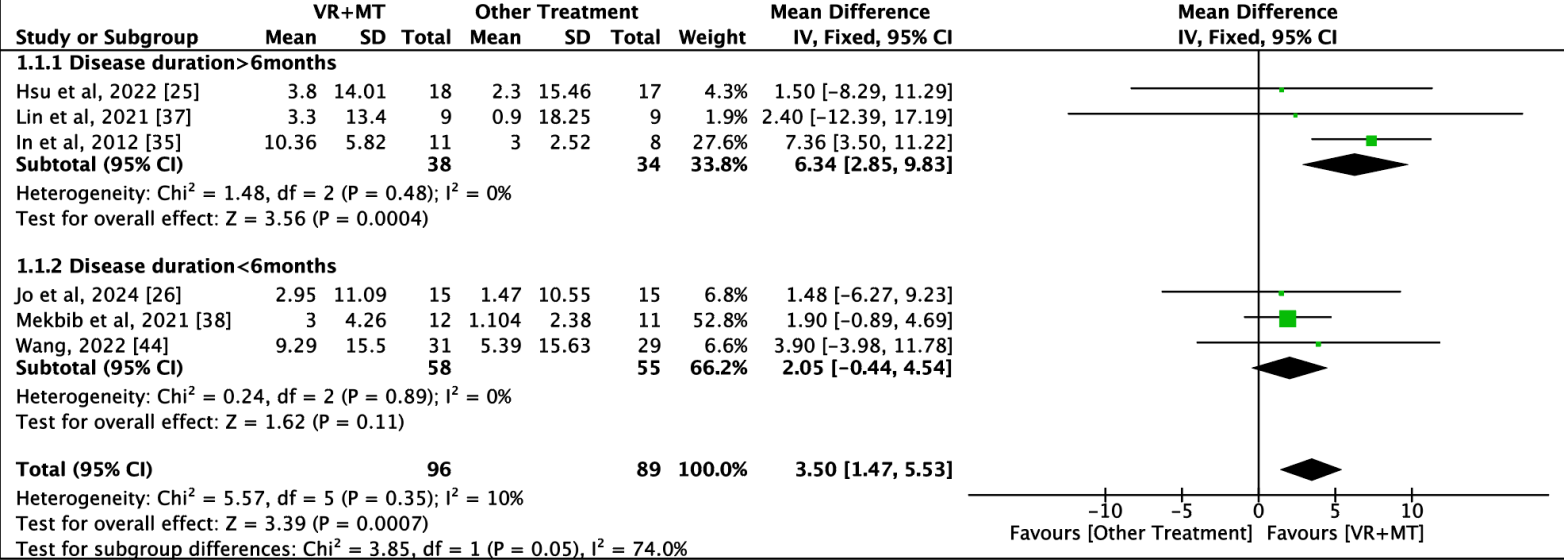
Forest plot for Fugl-Meyer Assessment–Upper Extremity (FMA-UE; total). IV: inverse variance; MT: mirror therapy; VR: virtual reality [25,26,35,37,38,44].

A total of 6 RCTs (43%) [25,26,35,37,38,44] with 185 participants (VR+MT: 96 and other treatment: 89) were included in the FMA-UE (total) analysis, and 3 RCTs (21%) [24,26,35] with 73 participants (VR+MT: 38 and other treatment: 35) were included in the MFT analysis. Compared with other treatments, the use of combined therapy was associated with significant improvements in FMA-UE (MD 3.50, 95% CI 1.47 to 5.53; *P*=.<0007) and MFT (MD 2.15, 95% CI 1.22 to 3.09; *P*<.00001). The forest plots are shown in Figures4  5. No publication bias was observed in the analysis.

**Figure 5.**
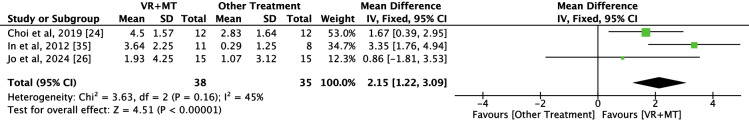
Forest plot for manual function test (MFT). IV: inverse variance; MT: mirror therapy; VR: virtual reality [24,26,35].

A review of 2 RCTs (14%) [25,37] indicates variable improvements across subdomains of the FMA-UE in patients with chronic stroke. Hsu et al [25] reported significant improvements in the shoulder, elbow, and forearm (*P*=.006), wrist (*P*=.002), hand (*P*<.001), and coordination (*P*<.001) subscores in the virtual reality-mirror therapy (VR-MT) group compared with baseline. In addition, the VR-MT group showed significantly greater improvement in the hand subscore (*P*=.01) compared to traditional MT. Complementarily, Lin et al [37] also found that the participants receiving VR-MT achieved greater improvement in the hand section of the Fugl-Meyer Assessment (*P*=.008) compared to the MT group. These findings collectively support the notion that VR-MT is particularly beneficial for enhancing performance in the hand subdomain of the FMA-UE.

### Effects on Hand Dexterity (BBT)

**Figure 6.**
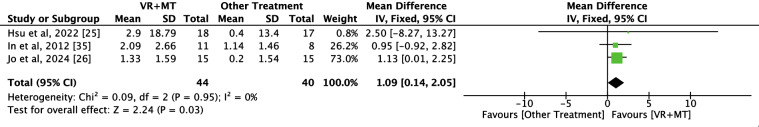
Forest plot for Box and Block Test. IV: inverse variance; MT: mirror therapy; VR: virtual reality [25,26,35].

Three RCTs (21%) [25,26,35] with 84 participants (VR+MT: 44 and other treatment: 40) assessed hand dexterity using the BBT. Compared with other treatments, the use of combined therapy was associated with significant improvements in hand dexterity (BBT; MD 1.09, 95% CI 0.14 to 2.05; *P*=.03). The forest plot is shown in Figure 6. No publication bias was observed in the analysis.

### Effects on Lower Extremity Motor Function (Fugl-Meyer Assessment–Lower Extremity)

Current evidence from 2 RCTs (14%) [39,43] indicates that VR combined with MT exerts a significant positive effect on LE motor recovery in stroke survivors, as reflected by improvements in the Fugl-Meyer Assessment–Lower Extremity (FMA-LE). In the study by Miclaus et al [39], the VR-MT group exhibited significantly greater improvements in FMA-LE scores, both within the group (*P*<.001) and in comparison to the control group receiving alternative therapy (*P*<.001). A similar trend was observed in Xu’s study [43], which reported statistically significant improvements in FMA-LE scores following VR-MT intervention (*P*<.01), and further demonstrated that VR-MT was significantly more effective than MT alone (*P*<.01). Taken together, these findings support the integration of VR-MT into LE rehabilitation programs as a promising strategy to enhance motor recovery in individuals post stroke.

### Effects on Dynamic Balance (Berg Balance Scale, Timed Up and Go Test, and Functional Reach Test)

Three RCTs (21%) [39,42,43] assessed the dynamic balance ability of patients with poststroke using the Berg Balance Scale (BBS), Timed Up and Go Test (TUGT), or Functional Reach Test (FRT). In a study by In et al [42], the effects of VR-MT were compared with those of a placebo intervention in patients with stroke. The results showed significant improvements in BBS scores in both the VR-MT and control groups postintervention, with the VR-MT group demonstrating significantly greater gains (*P*<.05). Compared to baseline, the VR-MT group exhibited significant improvements in both FRT and TUGT scores (*P*<.05), whereas no significant changes were observed in the control group. Furthermore, the VR-MT group demonstrated superior performance in both FRT and TUGT compared to the control group (*P*<.05). Similarly, an RCT conducted by Xu [43] found that patients with stroke receiving the combined therapy showed significant improvements, with BBS scores increasing (*P*<.01) and TUGT scores decreasing (*P*<.01) posttreatment. Both scores were significantly better than those of patients who received MT alone (*P*<.01). In addition, Miclaus et al [39] reported that the VR-MT intervention significantly improved FRT scores (*P*<.001), with outcomes significantly superior to those achieved through standard physiotherapy (*P*=.001). In conclusion, VR-MT demonstrates superior efficacy in improving dynamic balance compared to other interventions, underscoring its potential as an effective rehabilitation strategy for patients with stroke.

### Effects on Activity of Daily Living (Barthel Index and Functional Independence Measure)

Two studies (14%) [38,39] investigated the effect of a combination of VR and MT in improving ADL in patients with poststroke conditions. In the study by Mekbib et al [38], a fully immersive VR intervention combined with MT led to significant improvements in the Barthel Index (*P*=.011), indicating enhanced independence in ADL. However, no significant differences were observed when comparing the VR-MT group to the OT group (*P*=.193). In contrast, Miclaus et al [39] found that VR-MT resulted in nonsignificant postintervention changes in Functional Independence Measure scores relative to baseline (*P*≥.99). Overall, although the combination of VR and MT shows potential in improving ADL performance among patients with poststroke conditions, the current evidence remains limited and inconsistent.

### Effects on QoL (Short-Form 8 and 36-Item Short Form Survey)

As a result of measuring the QoL using Short-Form 8 in a study by Choi et al [24], a significant improvement was found in the conventional MT and VR-MT groups after the intervention (*P*<.05), with comparable efficacy. In a study by Sip et al [41], the VR-MT group demonstrated significant improvements in 36-Item Short Form Survey questionnaire scores (*P*=.001), which were notably superior to those of the control group (*P*<.05).

### Subgroup Analyses

Regarding the FMA-UE (total) outcome, significant improvements were observed in the disease duration >6 months subgroups (MD 6.34, 95% CI 2.85 to 9.83; *P*=<.001). However, no significant improvements were observed in the disease duration <6 months subgroups (MD 2.05, 95% CI −0.44 to 4.54; *P*=.11). The forest plot is shown in Figure 4. The findings of subgroup analyses are shown in Table 3.

**Table 3.** Findings of subgroup analyses according to different disease duration.

Outcome	Included studies, n	Heterogeneity test	Statistical model	MD^a^ (95% CI)	*z* score	*P* value
FMA^b^-UE^c^ (total)						
>6 months	3	Low	Fixed effects	6.34 (2.85 to 9.83)	3.56	.0004
<6 months	3	Low	Fixed effects	2.05 (−0.44 to 4.54)	1.62	.11

a MD: mean difference.

b FMA: Fugl-Meyer Assessment.

c UE: upper extremity.

### Sensitivity Analyses

**Figure 7.**
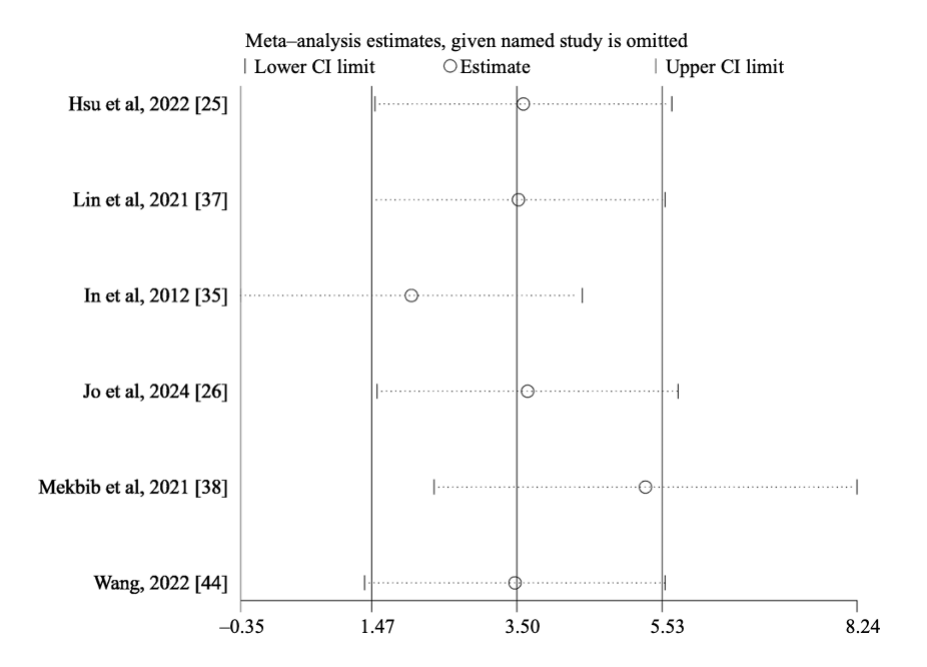
Sensitivity analyses for the total Fugl-Meyer Assessment–Upper Extremity (FMA-UE) score [25,26,35,37,38,44].

Sensitivity analyses, conducted by sequentially omitting individual studies, showed that the pooled effects for FMA-UE (total), MFT, and BBT remained statistically significant across most iterations, indicating a relatively high degree of robustness. The results of sensitivity analyses are shown in Figures7  8 and Multimedia Appendix 3.

**Figure 8.**
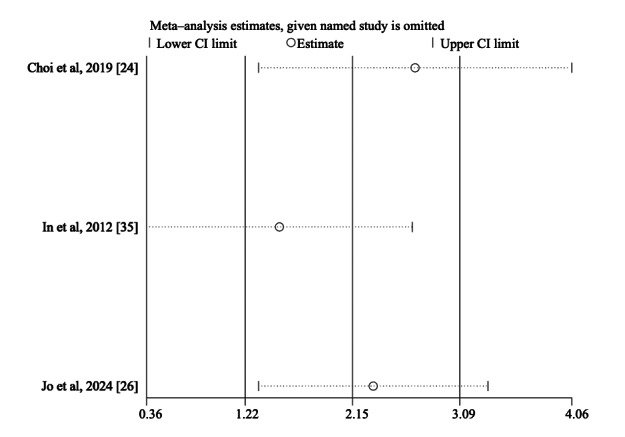
Sensitivity analyses for the manual function test (MFT) [24,26,35].

## Discussion

### Principal Findings

This systematic review investigated the effectiveness of VR-MT in treating patients post stroke using 14 RCTs. Among them, 6 studies [25,26,36-38,41] used IVR, while other studies [24,35,39,40,42-44] used NIVR. Various VR productions were used, including VR goggles from Oculus (Meta) [25,37,41], HTC Vive series productions (HTC) [36,38], Leap Motion Controller (Ultraleap) [24,25,37,38], Kinect sensor (Microsoft) [39], Pico head-mounted display (Pico) [26], and VR controller “etee” (TG0) [40]. Most studies [24-26,35-38,40-42,44,45] integrated VR with MT into a single intervention system, while only 2 studies [39,43] applied them separately.

The study by Lin et al [37] had the highest risk of bias. Performance bias, particularly in participant and personnel blinding, was the most common issue, highlighting a challenge in rehabilitation research where double-blinding is not feasible due to the nature of interventions [46]. Some studies suggest that double blinding may not be necessary for trials in real-life settings [47].

Although the pooled analyses showed a low degree of heterogeneity across outcomes (*I*²<50%), this consistency should be interpreted with caution. The included studies varied in terms of VR system types (immersive vs nonimmersive), intervention durations, frequencies, and patient characteristics such as age and stroke chronicity. Several factors may help explain the observed homogeneity. First, most studies used comparable outcome measures, such as FMA-UE and BBT, which reduce variability arising from measurement tools. Second, despite differences in specific implementations, all interventions shared core therapeutic components—namely, the integration of VR with MT—which may contribute to similar underlying mechanisms of action, particularly in activating the mirror neuron system and facilitating motor relearning. Third, the intervention durations and session frequencies across studies were within a relatively narrow range, which may further minimize variance. In addition, it is important to consider that the limited number of studies included in each analysis reduces the statistical power to detect between-study heterogeneity.

The different VR-MT formats (IVR vs NIVR and integrated vs sequential VR-MT) may affect interpretation and generalizability. IVR may offer more significant effects on neural plasticity, while NIVR might impact patient engagement differently. Integrated VR-MT, where both therapies are combined in a single session, may provide a more cohesive therapeutic experience, while sequential VR-MT may offer distinct benefits by alternating between the 2 therapies. Future studies should standardize these intervention formats to assess their clinical impact more reliably. In addition, heterogeneity may affect evidence certainty ratings, especially with varying VR platforms and MT methods.

It is also important to note that the comparator interventions among the included studies were heterogeneous, encompassing MT alone, OT, conventional rehabilitation, and sham interventions. This variability in control conditions may have introduced inconsistencies in treatment effects, potentially contributing to the observed heterogeneity in some pooled estimates. For instance, comparisons against sham interventions might yield larger effect sizes than those against active rehabilitation controls such as OT. While stratified meta-analysis by comparator type was not feasible due to the limited number of studies in each subgroup, this heterogeneity should be considered when interpreting the findings. The pooled estimates should therefore be understood as reflecting an average effect across diverse control conditions.

While moderate-quality evidence supports the combination of VR and MT as a beneficial approach for improving UE motor function and hand dexterity, the certainty of the evidence was downgraded due to factors such as imprecision and risk of bias. Specifically, in the subgroup analysis of patients with different disease durations, the evidence was downgraded to low quality due to wide CIs. Furthermore, the risk of bias in several studies, especially in terms of participant blinding, introduces additional uncertainty regarding the robustness of the findings. These limitations should be considered when interpreting the results, and future studies with larger sample sizes and more rigorous designs are needed to confirm these findings.

### Effects on UE Motor Function (FMA-UE and MFT)

Our study revealed significant improvements in UE motor function, as measured by FMA-UE and MFT. The Fugl-Meyer Assessment is a clinically validated, feasible, and effective method for evaluating motor function in people who have experienced a stroke [48]. The MFT comprises 4 items each for the shoulder and hand, thereby reflecting UE function poststroke [49], and there is evidence of strong correlations between MFT and FMA-UE [50,51]. One plausible explanation for these findings is the reversal of “learned paralysis.” Stroke-induced paralysis is primarily attributed to irreversible damage to the internal capsule. Swelling and edema of the white matter cause disruptions of cortical function, leading to a form of “learned paralysis” that persists even after the swelling and edema resolve. Mirror-induced visual feedback may create an illusion of normal movement in the paralyzed limb. This realignment of vision with motor intention might help reverse “learned paralysis” caused by disrupted sensorimotor loops post stroke [52]. Simultaneously, VR has been shown to induce cortical reorganization [53], which could effectively prevent the disruption of cortical function and reverse “learned paralysis.”

The second possible mechanism involves the activation of mirror neurons. Mirror neurons, located in the frontal and parietal lobes, may be activated during both action execution and observation and are involved in integrating multimodal inputs such as visual stimuli, motor commands, and proprioceptive feedback [54]. Following a stroke, residual mirror neurons may remain viable but functionally dormant, falling below the activation threshold [52]. MT, however, might provide congruent visual input that stimulates these neurons, thereby reactivating downstream motor pathways [55]. In addition, the VR environment could further activate key regions of the mirror neuron system, enhancing functional connectivity between these areas and the sensorimotor cortex [56].

The last explanation is that both VR and MT might facilitate motor relearning. Virtual and mirror environments provide visual feedback, which may aid in motor skill acquisition [57,58]. In addition, while MT has been validated as a beneficial rehabilitation strategy, it confines patients to a limited motor area. Conversely, VR offers a significantly larger space [59], allowing us to conceptualize its combined use as a more comprehensive and effective “mirror” for rehabilitation.

Beyond these mechanistic considerations, VR has also been consistently recognized in the rehabilitation literature for its ability to increase patient engagement and motivation, which are critical determinants of adherence and long-term functional outcomes. VR-supported protocols often incorporate gaming features and adaptive levels of challenge, which provide real-time feedback and sustain user interest [60-63]. These characteristics help patients avoid boredom or frustration, thereby enhancing concentration, training intensity, and adherence to therapy [64,65]. These established benefits may further explain why the combination of VR with MT yields superior improvements in upper limb function.

Nonetheless, the pooled effect size for the FMA-UE fell below the previously proposed minimal clinically important differences range of 4.25‐7.25 points for FMA-UE in patients with stroke [66], suggesting that these improvements should be interpreted with caution. The main reasons may include insufficient intervention dosage and follow-up, high patient heterogeneity, inadequate sensitivity of FMA-UE, and effect dilution due to a small sample size. Future high-quality RCTs with larger sample sizes, longer intervention and follow-up periods, and stratified designs (grouped by stroke severity or time since onset) are needed to verify whether it can make a clinical breakthrough at a meaningful level.

### Effects on Hand Dexterity (BBT)

Our study findings suggest that combined therapy can lead to enhancements in hand dexterity (measured by BBT). The BBT is a standardized assessment tool that measures unilateral gross manual dexterity and is extensively used in clinical settings across diverse populations, including older adults and individuals with neurological disorders [67]. Beyond the explanations previously provided, additional factors contribute to this outcome. In VR-supported MT, the interaction with virtual objects typically necessitates the application of manual motor skills to manipulate and move these objects, thereby engaging individuals in dexterity exercises.

### Subgroup Analysis of the Effects of Combined Therapy

Subgroup analysis revealed a trend based on disease duration: patients with poststroke duration >6 months exhibited greater improvements in UE motor function assessed by FMA-UE. This might be because chronic-phase patients rely more on subcortical and contralesional compensatory mechanisms [68,69]. In the chronic phase, motor recovery, being less dependent on corticospinal integrity [70], may benefit more from task-oriented, repetition-based interventions [71], which are enhanced by VR-MT protocols, since they provide enriched multisensory feedback and facilitate high-repetition training [57,72]. These findings underscore the importance of individualizing rehabilitation strategies based on poststroke duration to optimize functional outcomes.

### Limitations

Several limitations of this review should be acknowledged. The small sample sizes of individual studies may have limited the generalizability of the findings. In addition, several subgroup analyses were based solely on data from single studies, which further limited generalizability and complicated interpretation. Although presented for completeness, these results should be considered primarily descriptive or hypothesis-generating, rather than inferential.

Furthermore, the optimal duration and frequency of the combined VR-MT intervention could not be adequately evaluated due to the limited number of eligible studies. Beyond the assessed outcomes, the potential benefits of VR-MT for cognitive, speech, and swallowing functions remain unexplored, as these domains were not addressed in the included trials.

Critically, the lack of objective neurophysiological assessments—such as functional near-infrared spectroscopy, electromyography, or movement-related evoked potentials—in most studies hinders mechanistic understanding of the observed effects. This review also highlights a substantial gap in long-term follow-up data. The short duration of follow-up in most included RCTs precludes evaluation of sustained effects, thereby limiting understanding of the long-term clinical impact of VR-MT.

Finally, the feasibility of implementing VR-MT in routine clinical practice remains unclear and warrants further investigation. Potential barriers—such as equipment costs, limited accessibility in diverse health care settings, and patient adherence—have been infrequently addressed in the current literature, yet represent critical factors for real-world implementation.

These limitations should be considered when interpreting the findings of this systematic review and meta-analysis. Future research should prioritize these aspects to better inform clinical translation and implementation strategies.

### Conclusions

This systematic review and meta-analysis provide moderate-quality evidence that the combination of VR and MT yields statistically significant improvements in UE motor function and hand dexterity in patients after stroke. Nonetheless, the pooled effect size for the primary outcome (FMA-UE) fell below the established minimal clinically important difference threshold. Additional descriptive evidence points to potential benefits for lower extremity function, balance, and QoL, but these outcomes were not supported by meta-analyses and thus represent preliminary findings. Taken together, VR-MT may be considered a promising adjunctive rehabilitation strategy, though further large-scale, rigorously designed trials are needed to establish its true clinical value and long-term effectiveness.

## Supplementary material

10.2196/73142 Multimedia Appendix 1 Details of the search.

10.2196/73142 Multimedia Appendix 2 Results of the Grading of Recommendations, Assessment, Development, and Evaluation (GRADE).

10.2196/73142 Multimedia Appendix 3 Sensitivity analyses for the Box and Block Test.

10.2196/73142 Checklist 1 PRISMA checklist.

## Abbreviations

ADL: activities of daily living
BBS: Berg Balance Scale
BBT: Box and Block Test
FMA-LE: Fugl-Meyer Assessment–Lower Extremity
FMA-UE: Fugl-Meyer Assessment–Upper Extremity
FRT: Functional Reach Test
GRADE: Grading of Recommendations, Assessment, Development, and Evaluation
IVR: immersive virtual reality
LE: lower extremity
MD: mean difference
MFT: manual function test
MT: mirror therapy
NIVR: nonimmersive virtual reality
OT: occupational therapy
PICOS: population, intervention, comparison, outcome, and study design
PRISMA: Preferred Reporting Items for Systematic Reviews and Meta-Analyses
QoL: quality of life
RCT: randomized controlled trial
TUGT: Timed Up and Go Test
UE: upper extremity
VR: virtual reality
