# Supplementary material for: Combined Immersive and Nonimmersive Virtual Reality With Mirror Therapy for Patients With Stroke: Systematic Review and Meta-Analysis of Randomized Controlled Trials
Source: J Med Internet Res. 2025 Oct 10;27:e73142. doi: 10.2196/73142 (PMC12513685; doi:10.2196/73142)
Supplement: Multimedia Appendix 1 [file jmir-v27-e73142-s001.docx]

**Multimedia Appendix 1.** Details of the search

**PubMed (no date restrictions)**

| **#** | **Search terms** | **Results** |
| --- | --- | --- |
| 1 | ((((((((((((((((((((((((((((Stroke[MeSH Terms]) OR (Strokes[Title/Abstract])) OR (Cerebrovascular Accident[Title/Abstract])) OR (Cerebrovascular Accidents[Title/Abstract])) OR (CVA (Cerebrovascular Accident[Title/Abstract]))) OR (CVAs (Cerebrovascular Accident[Title/Abstract]))) OR (Cerebrovascular Apoplexy[Title/Abstract])) OR (Apoplexy, Cerebrovascular[Title/Abstract])) OR (Vascular Accident, Brain[Title/Abstract])) OR (Brain Vascular Accident[Title/Abstract])) OR (Brain Vascular Accidents[Title/Abstract])) OR (Vascular Accidents, Brain[Title/Abstract])) OR (Cerebrovascular Stroke[Title/Abstract])) OR (Cerebrovascular Strokes[Title/Abstract])) OR (Stroke, Cerebrovascular[Title/Abstract])) OR (Strokes, Cerebrovascular[Title/Abstract])) OR (Apoplexy[Title/Abstract])) OR (Cerebral Stroke[Title/Abstract])) OR (Cerebral Strokes[Title/Abstract])) OR (Stroke, Cerebral[Title/Abstract])) OR (Strokes, Cerebral[Title/Abstract])) OR (Stroke, Acute[Title/Abstract])) OR (Acute Stroke[Title/Abstract])) OR (Acute Strokes[Title/Abstract])) OR (Strokes, Acute[Title/Abstract])) OR (Cerebrovascular Accident, Acute[Title/Abstract])) OR (Acute Cerebrovascular Accident[Title/Abstract])) OR (Acute Cerebrovascular Accidents[Title/Abstract])) OR (Cerebrovascular Accidents, Acute[Title/Abstract]) | 214,301 |
| 2 | ((((((((((((Virtual Reality[MeSH Terms]) OR (Reality, Virtual[Title/Abstract])) OR (Virtual Reality, Educational[Title/Abstract])) OR (Educational Virtual Realities[Title/Abstract])) OR (Educational Virtual Reality[Title/Abstract])) OR (Reality, Educational Virtual[Title/Abstract])) OR (Virtual Realities, Educational[Title/Abstract])) OR (Virtual Reality, Instructional[Title/Abstract])) OR (Instructional Virtual Realities[Title/Abstract])) OR (Instructional Virtual Reality[Title/Abstract])) OR (Realities, Instructional Virtual[Title/Abstract])) OR (Reality, Instructional Virtual[Title/Abstract])) OR (Virtual Realities, Instructional[Title/Abstract]) | 9,599 |
| 3 | ((((((Mirror Movement Therapy[MeSH Terms]) OR (Mirror Movement Therapies[Title/Abstract])) OR (Movement Therapies, Mirror[Title/Abstract])) OR (Movement Therapy, Mirror[Title/Abstract])) OR (Therapies, Mirror Movement[Title/Abstract])) OR (Therapy, Mirror Movement[Title/Abstract])) OR (Mirror Therapy[Title/Abstract]) | 639 |
| 4 | ((#1) AND (#2)) AND (#3) | 10 |

**Embase (no date restrictions)**

| **#** | **Search terms** | **Results** |
| --- | --- | --- |
| 1 | 'cerebrovascular accident'/exp OR 'cerebrovascular accident' | 464,347 |
| 2 | 'accident, cerebrovascular' OR 'acute cerebrovascular lesion' OR 'acute focal cerebral vasculopathy' OR 'acute stroke' OR 'apoplectic stroke' OR 'apoplexia' OR 'apoplexy' OR 'blood flow disturbance, brain' OR 'brain accident' OR 'brain attack' OR 'brain blood flow disturbance' OR 'brain insult' OR 'brain insultus' OR 'brain vascular accident' OR 'cerebral apoplexia' OR 'cerebral insult' OR 'cerebral stroke' OR 'cerebral vascular accident' OR 'cerebral vascular insufficiency' OR 'cerebro vascular accident' OR 'cerebrovascular arrest' OR 'cerebrovascular failure' OR 'cerebrovascular injury' OR 'cerebrovascular insufficiency' OR 'cerebrovascular insult' OR 'cerebrum vascular accident' OR 'cryptogenic stroke' OR 'cva' OR 'insultus cerebralis' OR 'ischaemic seizure' OR 'ischemic seizure' OR 'stroke' OR 'thrombotic stroke' OR 'cerebrovascular accident' | 738,292 |
| 3 | #1 OR #2 | 738,292 |
| 4 | 'virtual reality'/exp OR 'virtual reality' | 38,272 |
| 5 | 'mirror therapy'/exp OR 'mirror therapy' | 1,060 |
| 6 | 'mirror feedback therapy' OR 'mirror movement therapy' OR 'mirror therapy (mt)' OR 'mirror visual feedback' OR 'mirror visual feedback (mvf) therapy' OR 'mirror visual feedback therapy' OR 'mirror visual feedback therapy (mvft)' OR 'mirror visual therapy' OR 'mvft (mirror visual feedback therapy)' OR 'mirror therapy' | 1,150 |
| 7 | #5 OR #6 | 11,50 |
| 8 | #3 AND #4 AND #7 | 81 |

**Scopus (no date restrictions)**

| **#** | **Search terms** | **Results** |
| --- | --- | --- |
| 1 | TITLE-ABS-KEY ( "Stroke" OR "Strokes" OR "Cerebrovascular Accident" OR "Cerebrovascular Accidents" OR "CVA (Cerebrovascular Accident)" OR "CVAs (Cerebrovascular Accident)" OR "Cerebrovascular Apoplexy" OR "Apoplexy, Cerebrovascular" OR "Vascular Accident, Brain" OR "Brain Vascular Accident" OR "Brain Vascular Accidents" OR "Vascular Accidents, Brain" OR "Cerebrovascular Stroke" OR "Cerebrovascular Strokes" OR "Stroke, Cerebrovascular" OR "Strokes, Cerebrovascular" OR "Apoplexy" OR "Cerebral Stroke" OR "Cerebral Strokes" OR "Stroke, Cerebral" OR "Strokes, Cerebral" OR "Stroke, Acute" OR "Acute Stroke" OR "Acute Strokes" OR "Strokes, Acute" OR "Cerebrovascular Accident, Acute" OR "Acute Cerebrovascular Accident" OR "Acute Cerebrovascular Accidents" OR "Cerebrovascular Accidents, Acute" ) | 639,735 |
| 2 | TITLE-ABS-KEY ( "Virtual Reality" OR "Reality, Virtual" OR "Virtual Reality, Educational" OR "Educational Virtual Realities" OR "Educational Virtual Reality" OR "Reality, Educational Virtual" OR "Virtual Realities, Educational" OR "Virtual Reality, Instructional" OR "Instructional Virtual Realities" OR "Instructional Virtual Reality" OR "Realities, Instructional Virtual" OR "Reality, Instructional Virtual" OR "Virtual Realities, Instructional" ) | 183,824 |
| 3 | TITLE-ABS-KEY ( "Mirror Movement Therapy" OR "Mirror Movement Therapies" OR "Movement Therapies, Mirror" OR "Movement Therapy, Mirror" OR "Therapies, Mirror Movement" OR "Therapy, Mirror Movement" OR "Mirror Therapy" ) | 1,053 |
| 4 | 1 AND 2 AND 3 | 96 |

**Cochrane Library (no date restrictions)**

| **#** | **Search terms** | **Results** |
| --- | --- | --- |
| 1 | MeSH descriptor: [Stroke] explode all trees | 17,550 |
| 2 | Cerebrovascular Stroke OR Cerebrovascular Accident OR Apoplexy OR CVA (Cerebrovascular Accident) OR Cerebral Stroke OR Apoplexy, Cerebrovascular OR Vascular Accidents, Brain OR Brain Vascular Accident OR Cerebrovascular Strokes OR Cerebrovascular Apoplexy OR Cerebrovascular Accidents OR Strokes, Cerebrovascular OR Vascular Accident, Brain OR Strokes OR Brain Vascular Accidents OR Strokes, Cerebral OR Stroke, Cerebral OR CVAs (Cerebrovascular Accident) OR Cerebral Strokes OR Stroke, Cerebrovascular OR Stroke, Acute OR Acute Cerebrovascular Accident OR Strokes, Acute OR Acute Strokes OR Cerebrovascular Accident, Acute OR Acute Stroke OR Acute Cerebrovascular Accidents OR Cerebrovascular Accidents, Acute | 48,537 |
| 3 | #1 OR #2 | 55,319 |
| 4 | MeSH descriptor: [Virtual Reality] explode all trees | 11,56 |
| 5 | Virtual Reality, Instructional OR Virtual Realities, Instructional OR Instructional Virtual Reality OR Reality, Instructional Virtual OR Educational Virtual Reality OR Reality, Educational Virtual OR Virtual Reality, Educational OR Virtual Realities, Educational OR Educational Virtual Realities OR Instructional Virtual Realities OR Realities, Instructional Virtual OR Reality, Virtual | 7,554 |
| 6 | #4 OR #5 | 7,564 |
| 7 | MeSH descriptor: [Mirror Movement Therapy] explode all trees | 35 |
| 8 | Therapies, Mirror Movement OR Mirror Movement Therapies OR Mirror Therapy OR Therapy, Mirror Movement OR Movement Therapy, Mirror OR Movement Therapies, Mirror | 1,431 |
| 9 | #7 OR #8 | 1,431 |
| 10 | #3 AND #6 AND #9 | 48 |
| 11 | Trials | 32 |

**Web of Science (no date restrictions)**

| **#** | **Search terms** | **Results** |
| --- | --- | --- |
| 1 | ((((((((((((((((((((((((((((TS=(Stroke)) OR TS=(Strokes)) OR TS=(Cerebrovascular Accident)) OR TS=(Cerebrovascular Accidents)) OR TS=(CVA (Cerebrovascular Accident))) OR TS=(CVAs (Cerebrovascular Accident))) OR TS=(Cerebrovascular Apoplexy)) OR TS=(Apoplexy, Cerebrovascular)) OR TS=(Vascular Accident, Brain)) OR TS=(Brain Vascular Accident)) OR TS=(Brain Vascular Accidents)) OR TS=(Vascular Accidents, Brain)) OR TS=(Cerebrovascular Stroke)) OR TS=(Cerebrovascular Strokes)) OR TS=(Stroke, Cerebrovascular)) OR TS=(Strokes, Cerebrovascular)) OR TS=(Apoplexy)) OR TS=(Cerebral Stroke)) OR TS=(Cerebral Strokes)) OR TS=(Stroke, Cerebral)) OR TS=(Strokes, Cerebral)) OR TS=(Stroke, Acute)) OR TS=(Acute Stroke)) OR TS=(Acute Strokes)) OR TS=(Strokes, Acute)) OR TS=(Cerebrovascular Accident, Acute)) OR TS=(Acute Cerebrovascular Accident)) OR TS=(Acute Cerebrovascular Accidents)) OR TS=(Cerebrovascular Accidents, Acute) | 451,808 |
| 2 | ((((((((((((TS=(Virtual Reality)) OR TS=(Reality, Virtual)) OR TS=(Virtual Reality, Educational)) OR TS=(Educational Virtual Realities)) OR TS=(Educational Virtual Reality)) OR TS=(Reality, Educational Virtual)) OR TS=(Virtual Realities, Educational)) OR TS=(Virtual Reality, Instructional)) OR TS=(Instructional Virtual Realities)) OR TS=(Instructional Virtual Reality)) OR TS=(Realities, Instructional Virtual)) OR TS=(Reality, Instructional Virtual)) OR TS=(Virtual Realities, Instructional) | 80,960 |
| 3 | ((((((TS=(Mirror Movement Therapy)) OR TS=(Mirror Movement Therapies)) OR TS=(Movement Therapies, Mirror)) OR TS=(Movement Therapy, Mirror)) OR TS=(Therapies, Mirror Movement)) OR TS=(Therapy, Mirror Movement)) OR TS=(Mirror Therapy) | 4,794 |
| 4 | #1 AND #2 AND #3 | 145 |
