# Supplementary material for: Combined Immersive and Nonimmersive Virtual Reality With Mirror Therapy for Patients With Stroke: Systematic Review and Meta-Analysis of Randomized Controlled Trials
Source: J Med Internet Res. 2025 Oct 10;27:e73142. doi: 10.2196/73142 (PMC12513685; doi:10.2196/73142)
Supplement: Multimedia Appendix 2 [file jmir-v27-e73142-s002.docx]

**Question:** VR+MT compared to other treatments for stroke

| **Certainty assessment** | | | | | | | | | | | | **№ of patients** | | **Effect** | | | | **Certainty** | | **Importance** | |  |  |
| --- | --- | --- | --- | --- | --- | --- | --- | --- | --- | --- | --- | --- | --- | --- | --- | --- | --- | --- | --- | --- | --- | --- | --- |
| **№ of studies** | | **Study design** | | **Risk of bias** | | **Inconsistency** | | **Indirectness** | | **Imprecision** | **Other considerations** | **VR+MT** | **other treatments** | **Relative (95% CI)** | | **Absolute (95% CI)** | |  |  |  |  |  |  |
| **FMA-UE (Total)** | | | | | | | | | | | | | | | | | | | | | |  |  |
| 6 | | randomised trials | | serious^a^ | | not serious | | not serious | | not serious | none | 96 | 89 | - | | MD **3.50 higher** (1.47 higher to 5.53 higher) | | ⨁⨁⨁◯ Moderate^a^ | |  | |  |  |
| **FMA-UE (Total) - Disease duration>6months** | | | | | | | | | | | | | | | | | | | | | |  |  |
| 3 | | randomised trials | | serious^a^ | | not serious | | not serious | | serious^b^ | none | 38 | 34 | - | | MD **6.34 higher** (2.85 higher to 9.83 higher) | | ⨁⨁◯◯ Low^a,b^ | |  | |  |  |
| **FMA-UE (Total) - Disease duration<6months** | | | | | | | | | | | | | | | | | | | | | |  |  |
| 3 | | randomised trials | | serious^a^ | | not serious | | not serious | | serious^b^ | none | 58 | 55 | - | | MD **2.05 higher** (0.44 lower to 4.54 higher) | | ⨁⨁◯◯ Low^a,b^ | |  | |  |  |
| **MFT** | | | | | | | | | | | | | | | | | | | | | |  |  |
| 3 | | randomised trials | | serious^a^ | | not serious | | not serious | | not serious | none | 38 | 35 | - | | MD **2.15 higher** (1.22 higher to 3.09 higher) | | ⨁⨁⨁◯ Moderate^a^ | |  | |  |  |
| **BBT** | | | | | | | | | | | | | | | | | | | | | | | |
| 3 | | randomised trials | | serious^a^ | | not serious | | not serious | | not serious | | none | 44 | 40 | | - | | MD **1.09 higher** (0.14 higher to 2.05 higher) | | ⨁⨁⨁◯ Moderate^a^ | |  | |

**CI:** confidence interval; **MD:** mean difference

#### Explanations

a. Lack of blinding of participants and personnel (performance bias)

b: Wide 95% CI of pooled effect
