# Supplementary figures and images for: Combined Immersive and Nonimmersive Virtual Reality With Mirror Therapy for Patients With Stroke: Systematic Review and Meta-Analysis of Randomized Controlled Trials
Source: J Med Internet Res. 2025 Oct 10;27:e73142. doi: 10.2196/73142 (PMC12513685; doi:10.2196/73142)

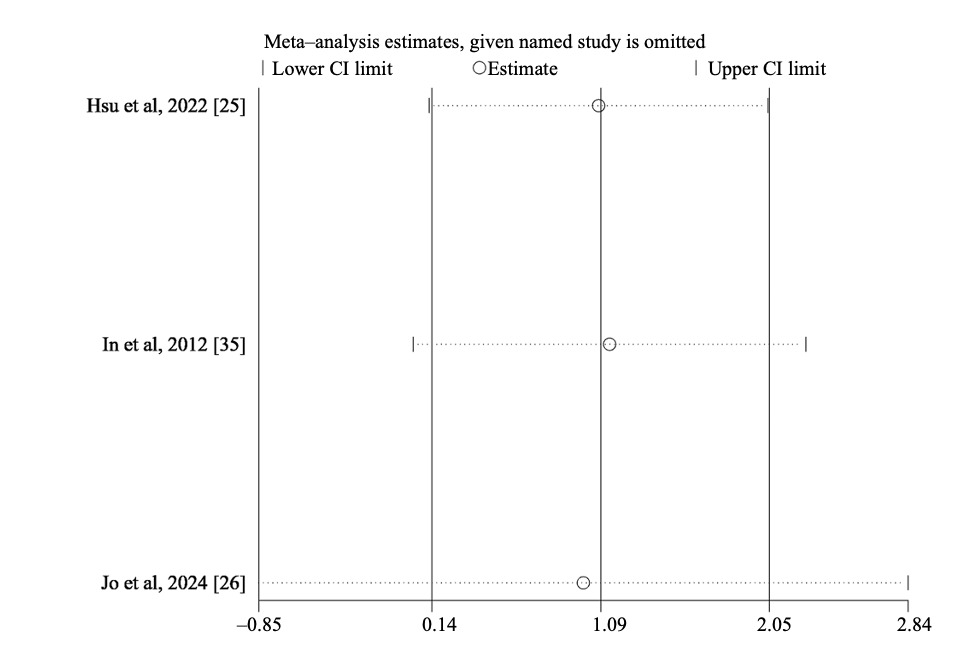

Supplement: Multimedia Appendix 3 [file jmir-v27-e73142-s003.png]
